# Mutations in Danish patients with long QT syndrome and the identification of a large founder family with p.F29L in *KCNH2*

**DOI:** 10.1186/1471-2350-15-31

**Published:** 2014-03-07

**Authors:** Michael Christiansen, Paula L Hedley, Juliane Theilade, Birgitte Stoevring, Trond P Leren, Ole Eschen, Karina M Sørensen, Anne Tybjærg-Hansen, Lilian B Ousager, Lisbeth N Pedersen, Ruth Frikke-Schmidt, Frederik H Aidt, Michael G Hansen, Jim Hansen, Poul E Bloch Thomsen, Egon Toft, Finn L Henriksen, Henning Bundgaard, Henrik K Jensen, Jørgen K Kanters

**Affiliations:** 1 Department of Clinical Biochemistry, Immunology and Genetics, Statens Serum Institut, Ørestads Boulevard 5, 2300S, Copenhagen, Denmark; 2 US/MRC Centre for Molecular and Cellular Biology, Division of Molecular Biology and Human Genetics, Department of Biomedical Science, University of Stellenbosch, Cape Town, South Africa; 3 The Heart Centre, Rigshospitalet, University of Copenhagen, Copenhagen, Denmark; 4 Department of Medical Genetics, University Hospital Rikshospitalet, Oslo, Norway; 5 Department of Cardiology, Ålborg Hospital, Aalborg University, Ålborg, Denmark; 6 Department of Clinical Biochemistry, Rigshospitalet, University of Copenhagen, Copenhagen, Denmark; 7 Department of Clinical Genetics, Odense University Hospital, Odense, Denmark; 8 Department of Molecular Medicine, Aarhus University Hospital Skejby, Aarhus, Denmark; 9 Department of Internal Medicine, Haderslev Hospital, Haderslev, Denmark; 10 Department of Cardiology, Gentofte Hospital, University of Copenhagen, Copenhagen, Denmark; 11 Center for Sensory-Motor Interaction, Aalborg University, Ålborg, Denmark; 12 Department of Cardiology, Odense University Hospital, Odense, Denmark; 13 Department of Cardiology, Aarhus University Hospital Skejby, Aarhus, Denmark; 14 Institute of Biomedical Science, University of Copenhagen, Copenhagen, Denmark; 15 Danish Arrhythmia Research Center (DARC), Copenhagen, Denmark

## Abstract

**Background:**

Long QT syndrome (LQTS) is a cardiac ion channelopathy which presents clinically with palpitations, syncope or sudden death. More than 700 LQTS-causing mutations have been identified in 13 genes, all of which encode proteins involved in the execution of the cardiac action potential. The most frequently affected genes, covering > 90% of cases, are *KCNQ1*, *KCNH2* and *SCN5A*.

**Methods:**

We describe 64 different mutations in 70 unrelated Danish families using a routine five-gene screen, comprising *KCNQ1*, *KCNH2* and *SCN5A* as well as *KCNE1* and *KCNE2.*

**Results:**

Twenty-two mutations were found in *KCNQ1*, 28 in *KCNH2,* 9 in *SCN5A,* 3 in *KCNE1* and 2 in KCNE2. Twenty-six of these have only been described in the Danish population and 18 are novel. One double heterozygote (1.4% of families) was found. A founder mutation, p.F29L in *KCNH2*, was identified in 5 “unrelated” families. Disease association, in 31.2% of cases, was based on the type of mutation identified (nonsense, insertion/deletion, frameshift or splice-site). Functional data was available for 22.7% of the missense mutations. None of the mutations were found in 364 Danish alleles and only three, all functionally characterised, were recorded in the Exome Variation Server, albeit at a frequency of < 1:1000.

**Conclusion:**

The genetic etiology of LQTS in Denmark is similar to that found in other populations. A large founder family with p.F29L in *KCNH2* was identified. In 48.4% of the mutations disease causation was based on mutation type or functional analysis.

## Background

Long QT syndrome (LQTS) is a genetic disease of the cardiac electrical system which presents clinically with palpitations, syncope and sudden death [1,2]. To date, more than 700 disease-causing mutations have been found in 13 genes [1-3] and the total number of mutations is probably larger [4]. All these genes are directly or indirectly involved in the execution of the cardiac action potential (AP) [2]. LQTS is a consequence of a prolongation of the repolarisation phase of the AP, caused by decreased activity of the repolarising inward K^+^-currents, *I*_ks_ and *I*_kr,_ or increased late activity of the outward depolarising Na^+^-current, *I*_Na_. The delayed repolarisation leads to the appearance of early after depolarisations (EADs), due to enhancement of the Na^+^/Ca^2+^-exchanger and the L-type Ca^2+^ channel [5]. These, together with increased refractoriness, may trigger malignant arrhythmias [2].

In Denmark, genetic diagnostics of LQTS has been performed since 1996. From 2006 the management of LQTS patients has followed national guidelines [6]. The genetic diagnostic work is centred in five University cardiology clinics and patients are offered a five-gene screen of the most frequently affected genes, *KCNQ1*, *KCNH2*, *SCN5A*, *KCNE1* and *KCNE2*. Initially, the screen was performed using single strand conformation polymorphism analysis (SSCP) of the coding regions of the genes with intronic amplification primers [7-9]. In recent years, the mutation analysis has been performed by bi-directional Sanger sequencing of coding regions and all previous patients have been reanalysed. Here we report the disease-causing mutations identified in Danish LQTS families during the preceding 15 years. Furthermore, as the evidence base for considering mutations disease-causing is not always clear [10], and rare variants which are not associated with disease are found in controls [11], we report our reasoning for considering the variants found in this cohort disease-causing. Finally, we compare the distribution of mutations with that found in other population studies.

## Methods

### Patients

The patients were 70 Danish LQTS probands from unrelated families where mutation screening in the five LQTS associated genes *KCNQ1*, *KCNH2*, *KCNE1*, *KCNE2* and *SCN5A* had led to the identification of a disease-causing mutation. LQT diagnosis was based on the clinical examination of patients, which was performed according to guidelines issued by the Danish Cardiology Society [6] by specialists in cardiology from Danish cardiology departments at Rigshospitalet, Skejby Hospital, Aalborg Hospital, Gentofte Hospital, Haderslev Hospital and Odense Hospital. All patients had a QT_C_ interval > 440 ms for men and 450 ms for women. All patients were Caucasian. A clinical description of patients identified through a Danish nationwide survey comprising 59 families (all contributing to the current survey) has recently been published [12].

### Mutation identification

Genomic DNA was extracted from EDTA-blood using the commercially available Maxwell™ 16 Blood DNA purification kit on the Maxwell^R^ 16 System (Promega Biotech AB, Nacka, Sweden). Genetic screening was performed by bi-directional sequencing of PCR amplified exons with associated flanking intronic regions. Primers sequences are available on request. A minor proportion of mutations were identified at genetic departments at Skejby University Hospital, Rigshospitalet as well as in Norway and the Netherlands using other, but similar, technologies. All probands had the coding regions of the five genes sequenced. All mutations were verified by sequencing a second amplified amplicon. A large proportion of patients were examined for large deletions in *KCNQ1*, *KCNH2*, *KCNE1*, *KCNE2* and a small part of *SCN5A* by multiplex-ligation-dependent amplification (MLPA) using the SALSA MLPA P114 kit (MRC-Holland, Amsterdam, The Netherlands).

### Mutation and protein nomenclature

Mutation nomenclature uses numbering with the A of the initiation codon ATG as +1 (http://www.hgvs.org/mutnomen), based on the following RefSeqs: NM_000218.2 (*KCNQ1*), NM_000238.2 (*KCNH2*), NM_000335.4 (*SCN5A*), NM_000219.3 (*KCNE1*) and NM_172201.1 (*KCNE2*). All mutations were checked using Mutalyzer. The protein nomenclature was that used in the recent mutation update on LQTS [2].

### Evaluation of sequence changes

Deletions, frameshift-, splice- and nonsense mutations were considered disease-causing if not found in controls. Concerning missense mutations, familial segregation was ascertained if possible, but nuclear family size was in all cases so small that it precluded a proper linkage analysis [12]. Instead it was ascertained that the family did not contain affected members that did not carry the family mutation. Conservation of residues across several species was examined. All genetic variants were evaluated in 182 randomly and anonymously collected blood donor controls (364 alleles). The frequency of identified variants was assessed using the Exome Variant Server (EVS) v.0.0.21. (http://evs.gs.washington.edu). It was established whether genetic variants had previously been associated with LQTS and whether functional analysis had been performed. The potential functional effect of changes in amino acid composition was assessed *in silico* using the prediction servers Polyphen-2 (http://genetics.bwh.harvard.edu/pph2/) [13], SIFT (http://sift.bii.a-star.edu.sg) [14], and MutationAssessor (http://www.ngrl.org.uk/Manchester/page/mutation-assessor) [15]

### Haplotyping *KCNH2*

**Figure 1:**
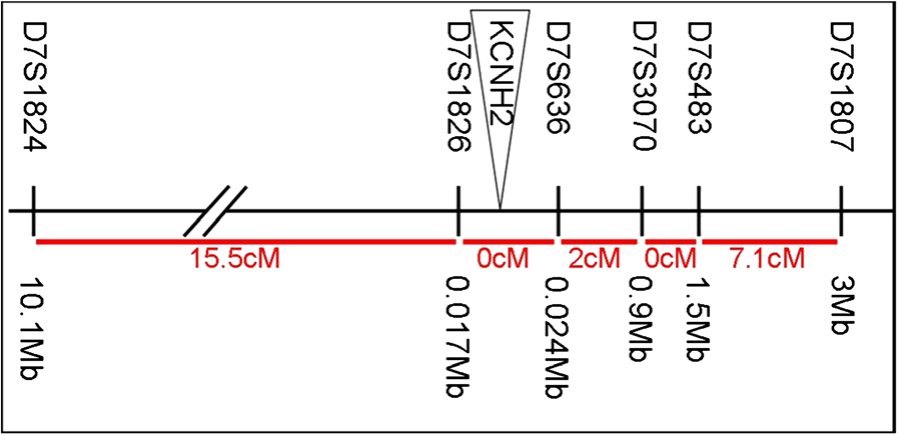
**Location of microsatellite markers used in haplotyping the p.F29L and p.K101E mutations in *KCNH2.***

Haplotyping was performed using the microsatellites D7S1824, D7S1826 up-stream of *KCNH2* and D7S636, D7S3070, D7S483 and D7S1803 downstream of *KCNH2* (Figure 1). CentiMorgan distances were obtained from the Map-O-Mat database for microsatellites (http://compgen.rutgers.edu/Mapomat/). PCR amplicons were generated using fluorescently end-labelled primers (available at NCBI UniSTS) at 0.4 μM per primer, per reaction. A loading mix of 0.5 μl amplicon, 9 μl HiDi formamide (Applied Biosystems, Foster City, CA, USA) and 0.5 μl 600LIZ size standard (Applied Biosystems) was prepared, and DNA products were electrophoresed on an ABI PRISM® 3100 Genetic Analyser. Data were analysed using ABI GeneMapper software v4.0 (Applied Biosystems).

## Results

### Mutation screening

The results of the mutation screening are summarized in Tables 1, 2, 3. In the 70 families we found 64 different mutations; 22 in *KCNQ1, 28* in *KCNH2*, 9 in *SCN5A*, 3 in *KCNE1* and 2 in *KCNE2*. All patients were heterozygous carriers of a single mutation, except one double heterozygous proband (1.4% of families), who carried p.R583G in *KCNQ1* and p.A93T in *KCNE1.* Twenty-six of the mutations have only been described in Danish LQTS patients (Tables 1, 2, 3), 18 have not been reported previously. Two mutations in *KCNH2*, p.F29L and p.K101E were found in 5 and 2 unrelated families, respectively. The MLPA analysis, applied to 65 of the probands, resulted in the identification of a single three exon (7-9) deletion, IVS6_IVS10del, in *KCNQ1.* None of the mutations were found in 384 control alleles.

**Table 1:** **Mutations identified in *KCNQ1***

**Gene**	**cDNA**	**Protein**	**Mutation type**	**Genomic region**	**Protein region**	**Phenotype**	**References**
***KCNQ1***	**NM_000218**	**NP_000209**					
	c.217C > A	p.P73T	Missense	Exon_01	N-term	RWS	[4,16]
DK	c.470T > G	p.F157C	Missense	Exon_02	S2	RWS	[17]
	c.572-576del	p.L191fs	Frameshift	Exon_03	S2-S3	RWS	[4,18]
	c.592A > G	p.I198V	Missense	Exon_03	S2-S3	RWS	[4]
	c.674C > T	p.S225L	Missense	Exon_04	S3-S4	RWS	[4,19]
	c.667C > T	p.A226V	Missense	Exon_04	S3-S4	RWS	[4,20]
	c.760G > A*	p.V254M	Missense	Exon_05	S4-S5	RWS	[4,20-26]
	c.817C > T*	p.L273F	Missense	Exon_06	S5	RWS	[4,20,21,25-28]
	c.905C > T	p.A302V	Missense	Exon_06	Pore	RWS	[4,22]
	c.944A > G*	p.Y315C	Missense	Exon_07	Pore	RWS	[4,19,20,22,29-31]
	c.1015-1017del	p.F339del	Deletion	Exon_07	S6	RWS	[32]
DK	IVS6_IVS10del	Unknown	Deletion	Exon_07-Exon_9	Unknown	RWS	This study
	c.1017-1019del	p.F340del	Deletion	Exon_07	S6	RWS	[4,20,25,33]
	c.1032G > A	p.A344sp	Splice-site	IVS_07	C-term	RWS	[4,22,25,26,34-37]
	c.1048G > A	p.G350R	Missense	Exon_08	C-term	RWS	[4,38]
DK	c.1087C > A	p.H363N	Missense	Exon_08	C-term	RWS	[2,26]
	c.1096C > T	p.R366W	Missense	Exon_08	C-term	RWS	[4,9,22,26,31]
	c.1202insC	p.P400fs	Frameshift	Exon_09	C-term	RWS	[4,20,39]
	c.1588C > T*	p.Q530X	Nonsense	Exon_12	C-term	RWS	[4,20,25,28,40,41]
	c.1747C > T*	p.R583C	Missense	Exon_15	C-term	RWS	[25,42]
DK	c.1747C > G	p.R583G	Missense	Exon_15	C-term	RWS	This study
	c.1748G > A	p.R583H	Missense	Exon_15	C-term	RWS	[38]

DK: Only seen in patients of Danish origin. *in vitro functional characterisation performed. RWS: Romano-Ward syndrome.

**Table 2:** **Mutations identified in *KCNH2***

**Gene**	**cDNA**	**Protein**	**Mutation type**	**Genomic region**	**Protein region**	**Phenotype**	**References**
***KCNH2***	**NM_000238**	**NP_000229**					
DK	c.65T > C	p.F22S	Missense	Exon_01	N-term	RWS	This study
	c.87C > A*	p.F29L	Missense	Exon_01	N-term	RWS	[4,26,43]
DK	c.88-90del	p.I30del	Deletion	Exon_01	N-term	RWS	This study
	c.221C > T	p.T74M	Missense	Exon_02	PAS	RWS	[4,38]
DK	c.234_241dupTGCCGCGC	p.A83fs	Frameshift	Exon_02	PAS	RWS	This study
DK	c.287T > C	p.I96T	Missense	Exon_02	PAS	RWS	[8]
DK	c.301A > G	p.K101E	Missense	Exon_02	PAS	RWS	[8,44]
DK	c.326T > C	p.L109P	Missense	Exon_03	PAS	RWS	This study
DK	c.446insC	p.R148fs	Frameshift	Exon_03	N-term	RWS	This study
	c.453delC	p.P151fs	Frameshift	Exon_03	N-term	RWS	[45]
	c.526C > T	p.R176W	Missense	Exon_04	N-term	RWS	[45]
DK	c.552-560del	p.G184-G188del	Deletion	Exon_04	N-term	RWS	This study
	c.1096C > T	p.R366X	Nonsense	Exon_05	N-term	RWS	[4,8]
DK	c.1199T > A	p.I400N	Missense	Exon_06	N-term	RWS	[8]
	c.1283C > T	p.S428L	Missense	Exon_06	S1-S2	RWS	[38]
DK	c.1286delC	p.S428fs	Frameshift	Exon_06	S1-S2	RWS	[46]
DK	c.1591-1671del	p.R531-L539del	Deletion	Exon_07	S4-S5	RWS	This study
	c.1682C > T	p.A561V	Missense	Exon_07	S5	RWS	[4,19,25,47]
DK	c.1714G > C	p.G572R	Missense	Exon_07	S5-pore	RWS	[48]
	c.1750G > A	p.G584S	Missense	Exon_07	Pore	RWS	[25,45]
	c.1862G > A	p.S621N	Missense	Exon_07	Pore	RWS	[8,49,50]
	c.1886A > G	p.N629S	Missense	Exon_07	Pore	RWS	[4,51]
	c.1898A > G	p.N633S	Missense	Exon_07	Pore-S6	RWS	[51]
DK	c.2111_2114dup	p.W705fs	Frameshift	Exon_08	C-term	RWS	This study
DK	c.2573T > C	p.I858T	Missense	Exon_10	C-term	RWS	This study
	c.2738C > T	p.A913V	Missense	Exon_12	C-term	RWS	[4,16]
	c.2768delC	p.P923fs	Frameshift	Exon_12	C-term	RWS	[52]
DK	c.3090-3102del	p.S1029fs	Frameshift	Exon_13	C-term	RWS	This study

DK: Only seen in patients of Danish origin. *in vitro functional characterization performed. RWS: Romano-Ward syndrome.

**Table 3:** **Mutations identified in *SCN5A*, *KCNE1 *** **or *KCNE2***

**Gene**	**cDNA**	**Protein**	**Mutation type**	**Genomic region**	**Protein region**	**Phenotype**	**References**
***SCN5A***	**NM_000335**	**NP_000326**					
DK	c.611+ 1G > A	Intronic splice	Splice-site	IVS5	D1-S3	RWS	This study
DK	c.955C > A	p.G319S	Missense	Exon_08	D1-S5-S6	RWS	[7]
	c.1018C > T	p.R340W	Missense	Exon_09	D1-S5-S6	RWS	[4]
DK	c.1141-3C > A	Intronic splice	Splice-site	IVS9	D1-S6	RWS	This study
	c.1231G > A	p.V411M	Missense	Exon_10	D1-S6	RWS	[4,16]
	c.4783T > A	p.F1595I	Missense	Exon_27	DIV-S3	RWS	[4]
DK	c.4786G > A	p.V1596M	Missense	Exon_27	DIV-S3	RWS	This study
DK	c.5354T > A*	p.L1785Q	Missense	Exon_28	C-term	RWS	This study
	c.6013C > G*	p.P2005A	Missense	Exon_28	C-term	RWS	[53]
***KCNE1***	**NM_000219**	**NP_000210**					
DK	c.179G > A	p.G60D	Missense	Exon_03	TM	RWS	This study
	c.226G > A*	p.D76N	Missense	Exon_03	Cyto	RWS	[4,25,52,54-56]
DK	c.277G > A	p.A93T	Missense	Exon_03	Cyto	RWS	This study
***KCNE2***	**NM_172201**	**NP_751951**					
	c.170T > C*	p.I57T	Missense	Exon_02	TM	RWS	[4,57-61]
	c.193G > A*	p.V65M	Missense	Exon_02	TM	RWS	[59]

DK: Only seen in patients of Danish origin. *in vitro functional characterization performed. RWS: Romano-Ward syndrome.

### *KCNQ1* mutations

The twenty-two mutations identified in *KCNQ1* seemed evenly spread out over the gene (Table 1). Only three of the mutations (p.F157C, IVS6_IVS10del and p.R583G) had not previously been described in other populations. The IVS6_IVS10del results, theoretically, either in the excision of a considerable part of the protein rendering it non-functional or in haploinsufficiency due to mRNA surveillance mechanisms [62], and is thus considered pathogenic. The remaining missense mutations all affect highly conserved residues and many missense mutations have been described in the S2-S3 and C-terminal regions of the protein, respectively [2]. The p.R583G affects a codon, where another missense mutation, p.R583C, has been shown to have an electrophysiological phenotype similar to that expected for a LQTS-associated mutation [42]. Among the 18 mutations previously seen in other populations, there were two frameshift mutations, two deletions, one splice-site mutation and one nonsense mutation. All of these must be expected to result in haploinsufficiency due to mRNA surveillance mechanisms. Of the remaining missense mutations, four had been electrophysiologically characterized *in vitro*, i.e. p.V254M [21], p.L273I [21,27,28], p.Y315C [43] and p.R583C [42]. Thus, only four out of 15 LQTS-associated missense mutations (27%) had established electrophysiological characteristics at the molecular level.

### *KCNH2* mutations

Twenty-eight mutations were identified in *KCNH2*. Fourteen of these were located in the N-terminus, and five of these were located in the Per-Arnt-Sim domain. Five were located in the C-terminus, only five mutations were located in the pore-region (Table 2). This is surprising, as the majority of previously identified LQTS associated mutations in *KCNH2* have been located in the pore region [2]. Fifteen of the mutations have only been described in the Danish population. Eight of the Danish specific mutations were deletions, or frameshift mutations. The remaining eight Danish-specific mutations were missense mutations and all involved conserved amino acid residues. Among the mutations found in other populations, two were frameshift mutations, one was nonsense and 10 were missense mutations. Only one of these missense mutations, p.F29L, had been demonstrated to have an *in vitro* electrophysiological effect compatible with LQTS [43], so the remainder were considered disease-associated based on the evolutionary conservation of the involved amino acid residue and the absence of the mutation among 364 control alleles as well as a previously reported association with LQTS. The observation of the association of these mutations with LQTS in the Danish population corroborates their role in the causation of LQTS. Finally, a large number of mutations in the regions affected by the mutations described here, have been associated with LQTS [2].

All the remaining missense mutations in *KCNH2,* except p.A913V, were conserved to the level of zebrafish. The p.A913V mutation changed an alanine into a valine, where the valine was found at the same codon in rodents. However, the mutation had previously been associated with LQTS [16] and in the absence of deviations from familial disease segregation, and the absence of other mutations in the five genes as well as of the mutation in 364 control alleles, it was considered disease-causing.

### *SCN5A* mutations

Nine mutations were identified in *SCN5A* (Table 3), five of which have only been described in the Danish population. Four mutations have not been reported before. Two mutations were intronic splice-site mutations and the remaining mutations were missense mutations located in the DI, DIV and C-terminal regions. This distribution is compatible with the one found when accumulating all known *SCN5A* LQTS-associated mutations [2]. The splice-site mutations were considered disease-causing because they are believed to result in aberrant mRNA splicing resulting in the synthesis of *SCN5A* ion channels with prolonged depolarisation contributing to delayed repolarisation. The splice mutations most likely do not result in haploinsufficiency as this would most likely give a Brugada syndrome phenotype [63]. One of the missense mutations, p.P2005A, has previously been associated with sudden infant death syndrome and shown to result in late persistent *I*_Na_ current, compatible with LQTS [53]. An *in vitro* electrophysiological analysis of the functional consequence of the p.L1785Q mutation has shown that it results in increased late persistent *I*_Na_ current, but also in a reduction of the total *I*_Na_ (Kanters et al., submitted), suggesting that the electrophysiological phenotype may be a combination of Brugada syndrome and LQTS, as previously described for *SCN5A* mutations [63], e.g. the p.E1784K mutation [64]. The remaining missense mutations interfere with conserved residues and have not been found in controls; further the mutations p.F1596I and p.V1597M, are located in the C-terminal part of *SCN5A*, where mutations causing LQTS are particularly frequent [2]. The rare polymorphism p.D1819N, known to be associated with increased QT-interval in normal individuals [65], was found in a single p.F29L family and not considered pathogenic, despite being previously reported as associated with LQTS [57] The C-terminally located mutations are likely to interfere with fast inactivation of the *I*_Na_ current [66]. Likewise, the remaining missense mutations, p.G319S, p.R340W and p.V411M, are located in the DI-S5-S6 region, a region with a high frequency of LQTS-associated mutations [2].

### *KCNE1* mutations

Three mutations were found in *KCNE1*, two of which, p.G60D and p.A93T, have only been found in the Danish population and have not been reported before. The p.A93T was found associated with p.R583G in *KCNQ1* in an isolated proband, where family data were not attainable. The p.G60D and p.D76N interfered with amino acid residues conserved in human, rat, mouse, cow and frog, whereas the alanine at residue 93 was only conserved in mouse, rat, cow and human. However, the N-terminus of frog minK is not conserved at all from amino acid residue 85 – 105 in the human sequence (corresponding to residue 80 – 95 in the frog minK sequence). However, the missense mutations p. Y81C, p.W87R, p.R98W, and p.P127T – as well as p.D76N, located in the cytoplasmic C-terminus of minK, have previously been associated with LQTS [2].

### KCNE2 mutations

Two mutations were found, p.I57T and p.V65M, both previously described in other populations as associated with LQTS [57,58] and shown to interfere with Kv7.1 function [59] in a way compatible with an association with LQTS. Both interfered with highly conserved residues and were not found in 384 control alleles.

### Disease causation

The association between a mutation and disease is of paramount importance when the mutation findings are used for cascade screening and clinical management as is the case in Denmark [12]. This is a particular problem when family data are not sufficient to establish linkage. As none of the mutations are present in the 364 control alleles and all but three (*SCN5A*:p.P2005A, *KCNE1*:p.D76N and *KCNE2*:p.I57T) were absent from the EVS, moreover, the three variants reported here occurred at frequencies < 1:1000, it is therefore unlikely that the variants reported here are polymorphisms. Frame-shift, splice-site- nonsense- or indel mutations represents 31.2% of the mutations identified in this cohort (Tables 1, 2, 3). Such mutations have a direct effect on the integrity of the polypeptide chain and are considered explanatory for LQTS. However, the remaining 68.8% are missense mutations, that need to be differentiated from the naturally occurring functionally insignificant non-synonymous variants in the same proteins. A classical method of supporting disease causality in LQTS is electrophysiological examination of mutated channel proteins to disclose a reduction in repolarising K^+^- current (*KCNQ1, KCNH2, KCNE1, KCNE2*) or late persistence of depolarising Na^+^- current (*SCN5A*) [2]. Such information, however, was only available for 22.7% of the missense mutations (Tables 1, 2, 3).

We used the prediction servers Polyphen-2, SIFT and Mutation Assessor to assess the significance of all the missense mutations for interference with protein function. The results are given in Table 4. As expected the majority of mutations where an electrophysiological assessment was available (p.V254M, p.L273F, p.Y315C, p.R583C in *KCNQ1*, p.F29L in *KCNH2*, p.L1785Q in *SCN5A*, p.D76N in *KCNE1*, p.I57T and p.V65M in *KCNE2*) were found to have an effect on protein function. One mutation, p.P2005A in *SCN5A* was not found to be disruptive of protein function, despite having been determined to cause functional impairment by *in vitro* electrophysiological assessment. This suggests that the sensitivity of these prediction servers is reasonable. The remaining, not functionally characterised, missense mutations, with the exception of *KCNQ1 -* p.P73T, p.R583G, p.R583H; *KCNH2*:p.A913V and *KCNE1*:p.A93T, were all found to be at least possibly disruptive of protein function.

**Table 4:** ***In silico *** **functional analysis of missense variants**

**Gene**	**Protein**	**Polyphen-2**	**SIFT**	**Mutation assessor**
*KCNQ1*	p.P73T	0	0	0
*KCNQ1*	p.F157C	0	1	2
*KCNQ1*	p.I198V	1	1	1
*KCNQ1*	p.S225L	1	1	1
*KCNQ1*	p.A226V	2	1	2
*KCNQ1*	p.V254M	2	1	2
*KCNQ1*	p.L273F	2	1	2
*KCNQ1*	p.A302V	2	1	2
*KCNQ1*	p.Y315C	2	1	3
*KCNQ1*	p.G350R	2	1	2
*KCNQ1*	p.H363N	1	1	2
*KCNQ1*	p.R366W	2	1	2
*KCNQ1*	p.R583C	1	0	1
*KCNQ1*	p.R583G	0	0	1
*KCNQ1*	p.R583H	0	0	1
*KCNH2*	p.F22S	2	0	2
*KCNH2*	p.F29L	0	1	2
*KCNH2*	p.T74M	2	1	2
*KCNH2*	p.I96T	1	1	2
*KCNH2*	p.K101E	0	1	3
*KCNH2*	p.L109P	1	0	2
*KCNH2*	p.R176W	2	1	0
*KCNH2*	p.I400N	2	1	2
*KCNH2*	p.S428L	0	0	2
*KCNH2*	p.A561V	2	1	2
*KCNH2*	p.G572R	2	1	2
*KCNH2*	p.G584S	1	0	0
*KCNH2*	p.S621N	1	1	3
*KCNH2*	p.N629S	2	1	2
*KCNH2*	p.N633S	1	0	1
*KCNH2*	p.I858T	1	1	2
*KCNH2*	p.A913V	0	0	0
*SCN5A*	p.G319S	0	0	2
*SCN5A*	p.R340W	1	1	0
*SCN5A*	p.V411M	2	1	3
*SCN5A*	p.F1595I	0	0	2
*SCN5A*	p.V1596M	1	1	2
*SCN5A*	p.L1785Q	2	1	3
*SCN5A*	p.P2005A	0	0	0
*KCNE1*	p.G60D	2	1	2
*KCNE1*	p.D76N	1	0	2
*KCNE1*	p.A93T	0	0	1
*KCNE2*	p.I57T	2	1	1
*KCNE2*	p.V65M	2	1	1

Polyphen-2 scores: 0: benign, 1 possibly damaging for function; 2: Probably damaging for function. SIFT scores: 0: Tolerated and 1: Not tolerated, Mutation Assessor scores; 0-1: no functional effect, 2-3: functional effect on protein function.

### The founder mutations in KCNH2

Two of the missense mutations, p.F29L [43] and p.K101E [44], were found in five and two “unrelated” families, respectively. Haplotype analysis, using six polymorphic microsatellite markers, spanning 24.6 cM, flanking the *KCNH2* gene at distances ranging from 10.1 Mb 3’ to 2.9 Mb 5’ as shown in Figure 1, demonstrated that both mutations were founder mutations (Additional file 1: Table S1).

### Population distribution of mutations

The distribution of Danish LQTS mutations is compared with four other large mutation surveys comprising verified LQTS patients in Table 5. The proportion of *KCNQ1* mutations seems to be lower in Danish patients, 34.3%, than in other populations, where the proportion of *KCNQ1* mutations ranged from 39.4% - 48.6%. This trend was not significant, however, using a Chi-Square test. The proportion of *KCNH2* mutations in Denmark is within the range seen in the other populations, whereas the proportion of patients with mutations in the three rarely affected genes, *SCN5A, KCNE1* and *KCNE2* is considerably higher, 23.7%, than seen in the other populations (range: 10.7% - 16.2%). The distribution of mutation types does not seem to differ substantially between the populations. Likewise, the frequency of compound heterozygosity was similar in the populations where it was established. In a Norwegian study no compound heterozygotes were convincingly demonstrated, but 18 cases of Jervell and Lange-Nielsen syndrome had been found [67].

**Table 5:** Distribution of mutations and mutation types in this study and four other large studies

	**This study**	**Berge et al **[67]	**Napolitano et al **[38]	**Tester et al **[16]	**Splawski et al **[25]
**Number of mutations**	64	37	233	211	177
*KCNQ1* (%)	34.3	42.6	48.6	41.7	39.4
*KCNH2* (%)	43.8	46.3	38.8	42.2	51.5
*SCN5A* (%)	14.1	9.3	10.1	15.2	6.1
*KCNE1* (%)	4.7	1.9	1.7	0.5	2.3
*KCNE2* (%)	3.1	0.0	0.7	0.5	2.3
**Mutation type**					
Missense (%)	68.8	64.9	72.0	75.0	72.3
Nonsense (%)	3.1	13.5	5.1	5.7	6.2
Deletion (%)	9.4	2.7	14.1	2.5	5.0
Frameshift (%)	14.1	13.5	6.1	11.4	9.6
Splice site (%)	4.7	5.4	2.7	4.3	6.7
Compound heterozygotes (%)*	1.4	0	3.9	5.4	n.a.

*Percentage of families.

## Discussion

We have identified 64 different mutations in 70 Danish LQTS families referred for five-gene screening. This is by far the highest number of different mutations identified per capita in any country and it amounts to approximately 1: 72.000 inhabitants. The signature of the genetics of LQTS in Denmark is that of many “private” mutations (Tables 1, 2, 3). With respect to this considerable inter-allelic and inter-genic variation, the Danish mutation spectrum is similar to that found in other populations (Table 5). However, there was a trend, albeit not significant, that the proportion of mutations in *KCNQ1* is reduced and the proportion of mutations in *SCN5A*, *KCNE1* and *KCNE2* correspondingly increased in the Danish LQTS patients. The same tendency is registered when comparing the Danish mutation spectrum to a collection of mutations identified in the five genes in persons referred for LQT testing, but where no knowledge on the clinical phenotype was available [4]. The relatively high frequency of mutations in the more rarely affected genes stresses the need of performing a five-gene screen when establishing the etiology of individual Danish LQTS families.

The patients described here were studied and collected over a long span of years, from 1996 – 2010. In this period the clinical picture of LQTS was better defined, the possibility of referral for genetic analysis increased and the indication for genetic analysis in LQTS cases was established in 2006 [6]. Furthermore, detailed clinical information was variable in quality, therefore it can not be excluded that, in some cases, other factors, e.g. structural heart disease or mixed phenotypes, might not have been identified and taken into account. Consequently, it is very difficult to establish a success rate for the genetic screening across this period. But it is probably comparable to the 70% reported from Norway in a much smaller collection of mutations [67]. Likewise, we cannot really state anything about the cost-effectiveness of including more genes in the basic screen or the suitability or cost-effectiveness of MLPA analysis of the five genes. A large proportion of cases, 65/70 had MLPA analysis performed in order to detect larger insertion/deletion mutations and a single case with a three exon deletion in *KCNQ1* was identified. However, considering the relatively low cost of MLPA analysis and the possibility to detect deletions that would have escaped classical Sanger sequencing, our data suggests that MLPA analysis – or more extensive methods for detection of minor structural abnormalities - should be part of the five gene screen as the frequency of deletions (1.4%) is comparable to that of KCNE mutations.

In general, disease causation was based on the identification of a mutation that; either resulted in a deletion or a frameshift, introduced a stop codon or disrupted correct splicing. In the case of missense mutations, the mutation should involve the exchange of a conserved amino acid and not be present in > 100 control alleles. If the mutation had previously been associated with LQTS this strengthened the argument for disease-causation. Functional assessment was not available for novel mutations. However, a large proportion, 48.4%, of mutations were classified as disease-causing as a consequence of the mutation type or the results of functional *in vitro* assessment. None of the mutations were present in Danish controls and three were found at very low frequencies in the EVS. The remaining missense mutations all, with five exceptions, were positive for disrupting protein function in one or several prediction servers. The prediction servers had a reasonable sensitivity, 9/10 functionally characterised mutations were correctly classified, but detailed analyses on larger datasets are necessary to establish specificity. Despite the promising performance of protein function interference prediction servers it will still be of great significance to be able to perform a functional analysis of identified novel variants.

Interestingly, this study and a subsequent survey of the cardiology clinics revealed that Andersen syndrome, despite being originally described in Denmark [68], as well as Jervell and Lange-Nielsen syndrome and Timothy syndrome patients, are not found in Danish cardiology clinics (Kanters, pers.com.).

The identification of a large *KCNH2* p.F29L founder family, comprising 7.1% of Danish LQTS families, is interesting, and the location of the family in the Northern part of Jutland, where it constitutes ca. 50% of affected families makes it easier to genotype patients from this part of Denmark. The p.F29L mutation has previously been found in North America [43] in a family of Northern European origin (Splawski, pers. com.). The mutation has been found to have an electrophysiological effect *in vitro* compatible with LQTS [43].

Each LQTS family has to be carefully examined as there is a risk of compound heterozygosity or digenic inheritance. Our data suggest that this risk is similar to that reported in other countries (Table 5). The clarification of the individual significance of either mutation in a family with a compound heterozygous index patient requires cascade screening to be performed and identification of carriers of the single mutations. In our experience this is rarely possible due to the small size of families [12].

The translation of molecular findings in LQTS patients into patient-specific clinical management decisions is difficult due to the low level of strict evidence, the complexity of the genetics, including the existence of genetic modifiers of phenotype [10]. Some of these problems may be alleviated if the use of patient-specific pluripotent stem cells turns out to give relevant information [69]. However, until such new approaches become routine, it is, with reference to the large role played by the use of previously reported information on disease-causation, important that all mutation findings and clinical as well as molecular follow-up of mutations are made available to the larger scientific community.

## Conclusions

The Danish spectrum of LQTS causing mutations is very similar to that of the rest of the world, even though the frequency of *KCNQ1* mutations seems relatively reduced and the proportion of mutations in rarely affected genes increased. There was a considerable proportion of novel mutations identified, but they were distributed on the genes largely as seen elsewhere. Despite a shortage of functional information and a long collection period nearly all mutations identified were reasonably classified as causative. The identification of a large founder family with p.F29L in *KCNH2* may become of importance for local patient management as well as studies into the prevention of sudden cardiac death in LQT2. Sharing of genotype and phenotype data as well as development of improved *in silico* predictions of functional consequences of mutations will improve the management of LQTS.

## Competing interest

The authors declare that they have no competing interests.

## Authors’ contributions

MC: participated in the design of the study, data acquisition, interpretation and drafting the manuscript; PLH: participated in data acquisition and data interpretation as well as critical revision of the manuscript; BS: participated in data acquisition, analysis and interpretation; JT: participated in data acquisition, analysis and interpretation as well as critical revision of the manuscript; TPL: participated in data acquisition, analysis and interpretation; OE: participated in data acquisition, analysis and interpretation; KMS: participated in data acquisition and analysis; ATH: participated in data acquisition, analysis and interpretation; LBO: participated in data acquisition, analysis and interpretation; LNP: participated in data acquisition, analysis and interpretation; RFS: participated in data acquisition, analysis and interpretation; FHA: participated in data acquisition and analysis; MGH: participated in data acquisition, analysis and interpretation; JH: participated in data acquisition, analysis and interpretation*; PEBT:* participated in data acquisition, analysis and interpretation; ET: participated in data acquisition, analysis and interpretation*; FLH:* participated in data acquisition, analysis and interpretation*; HB:* participated in data acquisition, analysis and interpretation*; HKJ:* participated in data acquisition, analysis and interpretation*; JKK*: participated in the design of the study, data acquisition and interpretation as well as critical revision of the manuscript. All authors read and approved the final manuscript.

## Pre-publication history

The pre-publication history for this paper can be accessed here:

http://www.biomedcentral.com/1471-2350/15/31/prepub

## Supplementary Material

Additional file 1: Table S1 Haplotyping of the p.K101E and p.F29L families. Click here for file
