# Supplementary material for: Mutations in Danish patients with long QT syndrome and the identification of a large founder family with p.F29L in KCNH2
Source: BMC Med Genet. 2014 Mar 7;15:31. doi: 10.1186/1471-2350-15-31 (PMC4007532; doi:10.1186/1471-2350-15-31)
Supplement: Additional file 1: Table S1 — Haplotyping of the p.K101E and p.F29L families. [file 1471-2350-15-31-S1.doc]

**Additional file 1: Table S1** Haplotyping of the p.K101E and p.F29 L families

|  | ped | ped | ped | Ped | Ped | ped | ped |
| --- | --- | --- | --- | --- | --- | --- | --- |
|  | 33 | 135 | 39 | 89 | 523 | 779 | 248_641_795 |
| D7S1824 | 0307 | 0104 | 08 | 06 | **02** | **02** | **02** |
| D7S1826 | **07** | **07** | **07** | **07** | **07** | **07** | **07** |
| KCNH2 | **101E** | **101E** | **29L** | **29L** | **29L** | **29L** | **29L** |
| D7S636 | **07** | **07** | **04** | **04** | **04** | **04** | **04** |
| D7S3070 | **08** | **08** | **06** | **06** | **08** | **06** | **06** |
| D7S483 | **06** | **06** | **08** | **08** | **07** | **08** | **08** |
| D7S1807 | 0108 | 06 | **02** | **02** | **04** | **02** | **02** |

The ancestral alleles are indicated blue or red text with regard to each mutation. Alleles are represented by approximate number of repeats.
